# Combination of quercetin and cisplatin enhances apoptosis in OSCC cells by downregulating xIAP through the NF-κB pathway

**DOI:** 10.7150/jca.31045

**Published:** 2019-07-25

**Authors:** Xin Li, Shu Guo, Xi-Kun Xiong, Bao-Ying Peng, Jun-Ming Huang, Mei-Fen Chen, Feng-Yan Wang, Jian-Ning Wang

**Affiliations:** 1 Guangdong Provincial Center for Disease Control and Prevention, 160 Qunxian Road, Dashi, Panyu District, Guangzhou, Guangdong Province, P.R. China, 511430; 2 State Environmental Protection Key Laboratory of Environmental Pollution Health Risk Assessment, South China Institute of Environmental Sciences, Ministry of Environmental Protection, Guangzhou, Guangdong Province, P.R. China, 510655; 3 Department of Oral and Maxillofacial Surgery, Guanghua School of Stomatology, Hospital of Stomatology, Institute of Stomatological Research, Sun Yat-sen University, Guangdong Provincial Key Laboratory of Stomatology, 56, Ling Yuan Xi Road, Guangzhou, Guangdong Province, P.R. China, 510055

**Keywords:** quercetin, cisplatin, apoptosis, NF-κB, xIAP

## Abstract

While cisplatin is a first-line chemotherapeutic drug commonly used to treat patients with oral squamous cell carcinoma (OSCC), the cisplatin-resistance poses a major challenge for its clinical application. Recent studies have shown that quercetin, a natural flavonoid found in various plants and foods possesses an anti-cancer effect. The following study examined the combined effect of quercetin and cisplatin on OSCC apoptosis *in vitro* and *in vivo* (using a mice tumor model). We found that quercetin promotes cisplatin-induced apoptosis in human OSCC (cell lines Tca-8113 and SCC-15) by down-regulating NF-κB. Pretreatment of cancer cells with quercetin inhibited the phosphorylation Akt and IKKβ, and led to the suppression of NF-κB and anti-apoptotic protein xIAP. In addition, we observed that the pretreatment of cancer cells with quercetin improves extrinsic and intrinsic apoptosis by activating caspase-8 and caspase-9, respectively. Our *in vivo* data also indicated that the combination of quercetin and cisplatin may inhibit the xenograft growth in mice. To sum up, our results provide a new evidence for the application of quercetin and cisplatin in OSCC therapy.

## 1. Introduction

Head and neck squamous cell carcinoma (HNSCC) is the sixth most common cancer worldwide. Oral squamous cell carcinoma (OSCC) accounts for over 40% of all HNSCC. In addition, more than 90% of oral malignancies are OSCC, with a yearly incidence of approximately 500,000 cases 1. Currently, the pathogenesis of OSCC remains unclear, and the therapeutic effect is not satisfactory. Except standard surgery and radiotherapy, chemotherapy has an important role in the treatment of OSCC. Cisplatin, also known as cis-diamminedichloroplatinum (II) (CDDP) is a first-line chemotherapeutic drug commonly used to treat patients with OSCC. It directly acts on DNA to form DNA adducts 2 by inducing cell death. However, genetic and epigenetic alternations brought by the drug may induce resistance to therapy.

NF-κB-induced cisplatin resistance has been previously reported in OSCC therapy 3, 4. The NF-κB transcription factor family in mammals is composed of five members, including RelA/p65, p105/p50, p100/p52, c-Rel, and RelB subunits. These form different dimers that have the function of transactivating target genes by binding to the κB enhancer. The p50/65 heterodimer that is found in almost all cells, is the most abundant among Rel dimers. In canonical NF-κB pathway, the p50/p65 dimers are sequestered in the cytoplasm by interacting with IκBα. IκBα is positively regulated by the IκB kinase (IKK) complex that phosphorylates IκBα protein and induces its degradation, and in turn releases NF-κB in the nucleus. Once in the nucleus, NF-κB binds to target DNA sequences and regulates the expression of genes related to cell survival, such as Bcl-2, Bcl-xl, Mcl-1, and xIAP 3.

Currently, flavonoids are considered as the ideal candidates for cancer chemoprevention 5, 6. Quercetin (3, 4, 5, 7-pentahydroxyflavone) is a flavonoid that is widely distributed in fruits and vegetables, including apples, blueberries, broccoli, grape, leek, lettuce, onion and tomato. Since it has the ability to modulate cell proliferation, survival and differentiation, as well as the ability to target key molecules responsible for tumor cell properties, it has been proposed as anticancer agent 7, 8.

The present study aimed to evaluate the combinative effects of cisplatin and quercetin on human OSCC *in vitro* and *in vivo*. Our findings suggested that quercetin promoted cisplatin-mediated apoptosis through the Akt-IKKβ-NF-κB-xIAP axis in OSCC and highlighted xIAP as a potential target for the OSCC therapy.

## 2. Materials and methods

### 2.1 Reagents and chemicals

Quercetin and cisplatin were purchased from Sigma-Aldrich (St. Louis, MO, USA). Caspase inhibitor z-VAD-fmk was purchased from Bio Mol. Antibodies against caspase-3, caspase-8, caspase-9, xIAP, IκBα, α-tubulin, and GAPDH were purchased from Cell Signaling Technology (Boston, USA). The antibody against cytochrome c was acquired from Epitomics (Burlingame, USA) and the antibody against PARP was purchased from BD Company (Franklin, USA). Goat anti-mouse IgG, FICT conjugated, was obtained from CW BIO (Beijing, China). Hoechst33342 was purchased from GenMed (Boston, USA). The secondary antibodies (horseradish peroxidase-conjugated goat anti-mouse IgG and goat anti-rabbit IgG) and the enhanced chemiluminescence substrate were purchased from BIO-RAD (Berkeley, USA). All other common chemicals were obtained from Sigma-Aldrich (St. Louis, MO, USA).

### 2.2 Cell culture and treatments

Two human oral squamous cell carcinoma cell lines, Tca-8113 and SCC-15, were obtained from Guanghua School of Stomatology, Sun Yat-Sen University (Guangzhou, China) and American Type Culture Collection (ATCC, Manassas, VA), respectively. All cells were cultured in DMEM medium (Gibco), supplemented with penicillin (100 units/ml), streptomycin (100 μg/ml), 10% fetal bovine serum (Gibco), and 5%, in a humidified atmosphere containing 5%CO_2_/95% air at 37ºC. According to different experiment designs, cells were plated with 5×10^4^ cells/mL and treated with cisplatin, quercetin and combined treatment (pretreatment with quercetin for 2 h followed by cisplatin), respectively; untreated cells were used as controls.

### 2.3 Measurement of cell apoptosis

The cells undergoing apoptosis were evaluated by chromatin condensation, nuclear shrinkage, and formation of apoptotic bodies. Briefly, cancer cells were first stained with Hoechst 33342 for 20 min and were then visualized using the inverted fluorescence microscope. Cell death was quantified by counting the percentage of cells with condensed nuclei among 200 randomly selected cells.

### 2.4 Colony formation assay

Cancer cells were plated on six-well plates (5,000 cells/well) for 24 h followed by various treatments. After three weeks, the survival clones were stained with 0.5% crystal violet for 1 h, and they were photographed with digital camera.

### 2.5 Lentivirus production and Transfection experiments

The human xIAP coding region was cloned into GV358 EGFP vector (Shanghai Genechem, China), packaged in plasmids and then transfected into 293T cells. Supernatant was harvested after 48 h and was then concentrated to the final lentivirus-xIAP (LV-XIAP). Stable transfection of Tca-8113 and SCC-15 cells was performed with virus LV-XIAP or CON238 vacant vector, using enhanced infection solution (Eni. S.) and polybrene (Shanghai Genechem Co. Ltd, China) according to the manufacturer's instructions. Then Tca-8113 and SCC-15 cells transfected with LV-XIAP or CON238 were treated with combined treatment (quercetin+cisplatin) to observe the cell death. Cell lysis was performed for protein analysis.

### 2.6 Western blotting analysis

After designated treatments, cancer cells were collected and washed in fresh PBS. Cell pellets were then lysed in RIPA buffer (CST) with a protease/phosphatase inhibitor cocktail (CST) according to the manufacturer's instructions. Aliquots of the lysates were subjected to SDS-PAGE and proteins in the gel were transferred to a polyvinylidene-fluoride membrane (Bio-Rad, Berkeley, USA). After blocking with 5% nonfat milk in TBST [10 mM Tris-HCl (pH7.5), 100 mM NaCl, and 0.05% Tween20], the membrane was probed with various antibodies and was developed with enhanced chemiluminescence (Bio-Rad, Berkeley, USA), using a CHEMI-SMART 3000 image station (VILBER-LOURMAT).

### 2.7 Preparation of cytosolic and nuclear extracts

Both the nuclear and cytosolic protein extracts were prepared using NE-PER Nuclear and Cytoplasmic Extraction Reagents (Thermo Scientific, USA), according to the manufacturer's instructions. Briefly, after treatment, cells were collected, washed, and re-suspended in ice-cold CER I buffer. After incubation on ice for 10 min, ice-cold CER II was added to the cell suspension, mixed and incubated for 2 min. The cytosolic extracts were collected after cells were centrifuged at 16,000g for 5 min. The nuclear pellets were then re-suspended in ice cold NER, and incubated for 40 min with vortexing for 15 s every 10 min. The nuclear extracts were collected after centrifugation (16,000g for 10 min, 4 ℃). Protein concentration was quantified using a Bio-Rad protein assay kit.

### 2.8 Immunofluorescence

Tca-8113 and SCC-15 cells were seeded in 24-well chamber slides for 24 h before treatment. After treatment, cells were washed with PBS and fixed in 3% paraformaldehyde for 1 h. After being permeabilized for 2min with 0.2% CHAPS in PBS, cells were blocked in 2% BSA with 0.2% Tween-20 for 30 min and further incubated with anti-p65 antibody at 48℃ overnight. After being washed with PBS (+0.2% Tween-20), cells were incubated with anti- anti-mouse FITC secondary antibody for 1 h. Cells were then analyzed under a Cellomics Toxinsight Reader (Thermo). The intensity of the p65 nuclear staining was measured in 49 fields.

### 2.9 RNA extraction and RT-PCR

Total RNA was isolated from cancer cells with designed treatment using PureLink RNA Mini Kit (Ambion). RT-PCR amplifications were performed using OneStep RT-PCR Kit (QIAGEN) and were carried out using Stratagene M3000 Sequence Detection System (Bio-rad, USA). RT-PCR conditions were 50 °C for 30 min, 95 °C for 15 min, followed by 94°C for 30 s, 55 °C for 30 s and 72 °C for 40 s, for 36 cycles. Sequences of primers used for RT-PCR were the following: xIAP mRNA, 5'-AAATAATGACTGCCCTGTTTCTG-3' (forward) and 5'- ATTGAACTACCTACCTGACTTGG -3' (reverse); GAPDH, 5'-GAAGGTGAAGGTCGGAGTC-3' (forward) and 5'-GAAGATGGTGATGGGATTTC-3' (reverse).

### 2.10 *In vivo* Xenograft

Balb/c male nude mice, 5-week-old, weighing 20-25 g, were obtained from Animal Resources Centre of Sun YAT-SEN University (Quality Certificate Number: 44008500011757) or Southern Medical University (Quality Certificate Number: 44002100009799) (Guangzhou, China). All the animals were housed in an environment with temperature of 22 ± 1 ºC, relative humidity of 50 ± 1% and a light/dark cycle of 12/12 hr. All animal studies (including the mice euthanasia procedure) were done in compliance with the regulations and guidelines of Guangdong Provincial Central for Disease Control and Prevention institutional animal care and conducted according to the AAALAC and the IACUC guidelines.

A total of 1×10^7^ Tca-8113 cells (in 100 μL PBS) were subcutaneously injected in the right flank. One week after inoculation, mice bearing tumors were randomly assigned to four experimental groups (six mice per group): vehicle control group (20% propanediol), quercetin group (50 mg/kg body weight), cisplatin group (2 mg/kg body weight), and combination group (quercetin 50 mg/kg body weight + cisplatin 2 mg/kg body weight). In this xenograft experiment, cisplatin was dissolved in normal saline (NS) and quercetin was dissolved in 20% propanediol solution prepared by NS. The doses of quercetin and cisplatin we used were significantly lower than the drug concentration used in other studies (ranging between 3 and 6 mg/kg). Our preliminary study indicated that both cisplatin (2 mg/kg) and quercetin (50 mg/kg) were not toxic to the mice (unpublished data). Quercetin was intraperitoneally injected (ip) every day for three weeks. In the combination group, quercetin was administrated for one week followed by cisplatin ip injection three times per week (every Monday, Wednesday, and Friday). Tumor size was measured every three days using an electronic caliper, and the tumor volume and the relative tumor volume (RTV) were calculated using the formula *V= (a^2^×b)/2* and *RTV*=*V_t_/V_0_*, where a is longer axis diameter, b is shorter axis diameter, V_0_ is the starting volume (before tretemnt) and V_t_ is the end volume (after experiment). The relative tumor proliferation rate (%) was measured using the following formula*:*

 (%)=RTV(experiment group)/RTV(vehicle control)×100%.

After three weeks, all mice were sacrificed. The tumors were isolated, and tumor weight and size were measured. Then, excised tumor tissues were lysed for protein or mRNA analysis.

### 2.11 Statistical analyses

All numerical data were presented as mean ± standard deviation (SD) from at least three independent experiments. The differences among distinct groups were evaluated using a t-test or a one-way ANOVA with LSD's test or Dunnett T3 test (SPSS 13.0). A P value <0.05 was considered statistically significant.

## 3. Results

### 3.1 Combination of quercetin and cisplatin promote human oral squamous cells apoptosis

**Figure 1:**
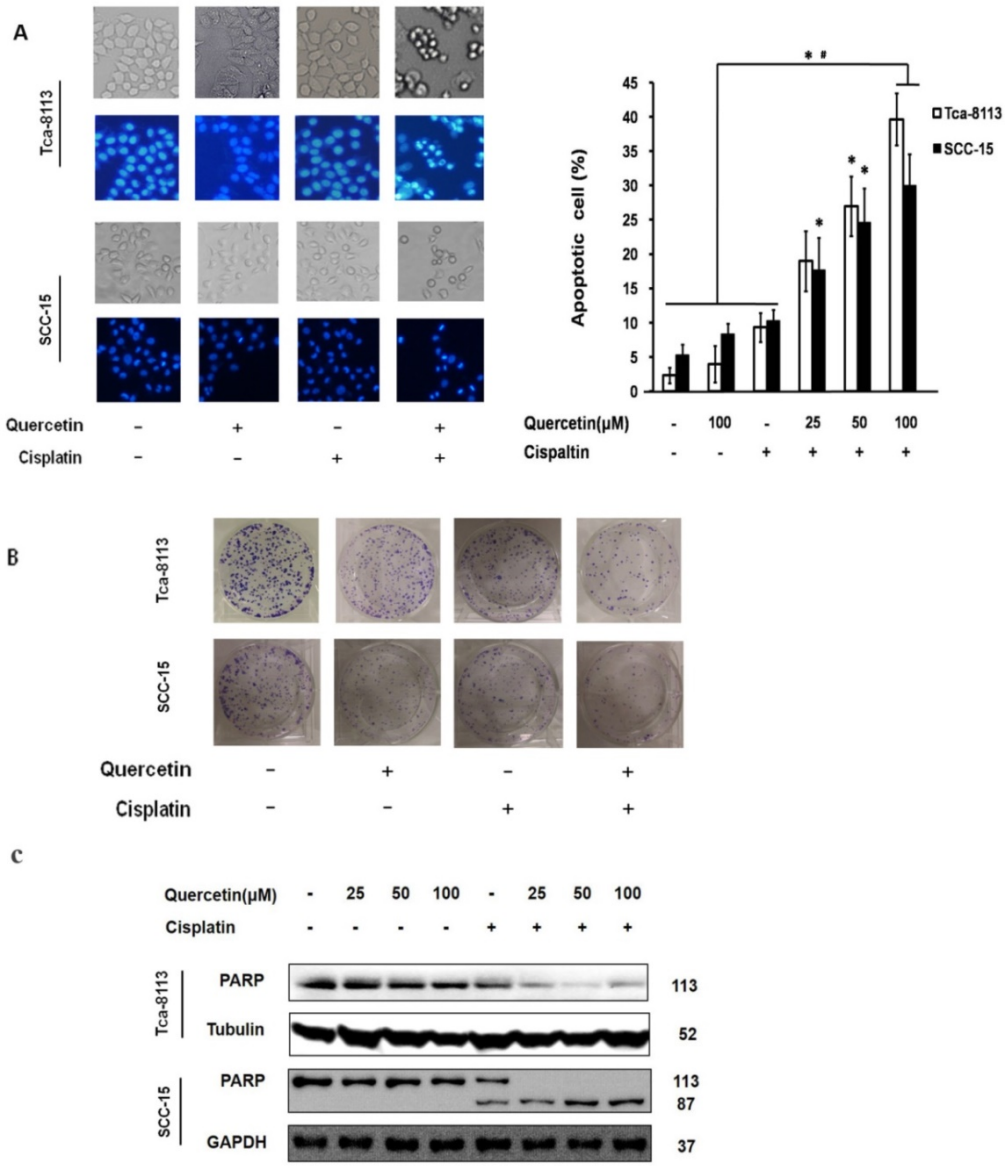
** Cotreatment of quercetin and cisplatin enhances oral squamous cell lines apoptosis (A)** Nuclear condensation in apoptotic cells detected by Hochest 33342 staining. Tca-8113 and SCC-15 cells were pretreated with quercetin (25, 50, 100 μM) for 2 h, followed by the addition of cisplatin (5 μg/ml) for another 24 h. The result was presented as the percentage of cells with evident nuclear condensation in 200 randomly selected cells. Representative images were photographed using normal light microscope (upper panel); cells with Hochest 33342 staining were visualized under an inverted fluorescence microscope (lower panel). **(B)** Colony formation assay. Tca-8113 and SCC-15 cells were pretreated with quercetin for 2 h; cells were then treated with or without the addition of cisplatin (5 μg/ml) for 6 h. The photos of colony formation were taken after 3 weeks. **(C)** Tca-8113 and SCC-15 cells were pretreated with quercetin (25, 50, 100 μM) for 2 h, followed by cisplatin (5 μg/ml) for another 24 h. Cell lysates were collected and subjected to Western blot analysis for the detection of PARP. The content of tubulin was used as a loading control. *p<0.05 vs untreated control; ^#^p <0.05 vs quercetin alone and cisplatin alone.

According to previous study, cisplatin-induced cell death has shown to be a relatively slow process that normally requires 2-3 days 9. In the present study, no significant cell death was observed when treating Tca-8113 and SCC-15 cells with cisplatin alone (5 μg/mL) for 24 h. Nevertheless, when cells were pretreated with quercetin (25, 50 and 100μM) for 2 h followed by cisplatin treatment (5 μg/mL) for 24 h, both cell lines underwent dramatic increase of apoptotic cell death in a dose-dependent manner (**Figure 1A**). Additionally, we found that the colony formation was obviously suppressed after co-treatment with quercetin (100μM) and cisplatin (5 μg/mL) for 6 h (**Figure 1B**). Furthermore, the expression of pro-protein of PARP and its active cleavage product were detected in both cell lines after treatment (**Figure 1C**).

### 3.2 A caspase cascade is involved in cell apoptosis activation induced by quercetin and cisplatin

**Figure 2:**
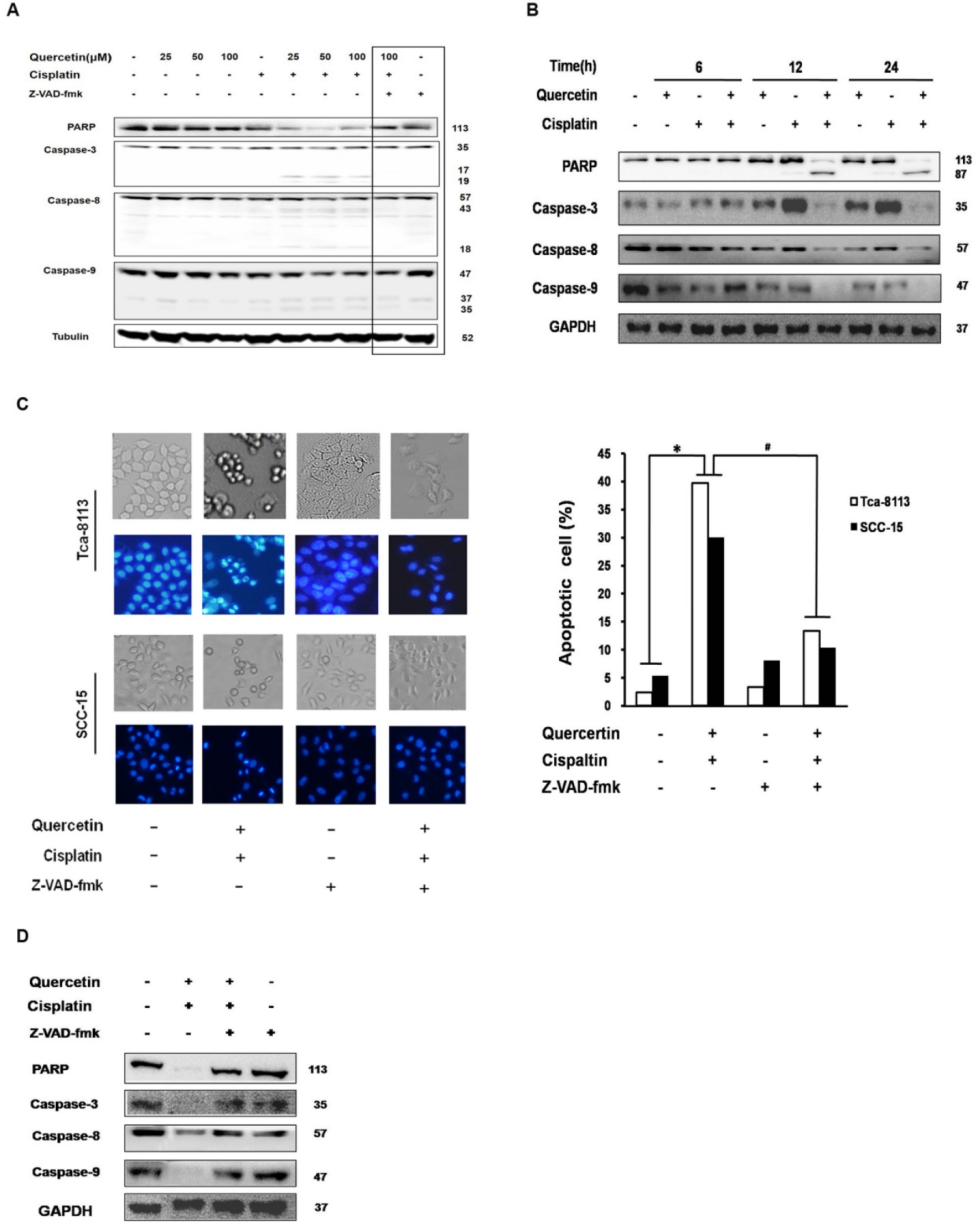
** Apoptosis enhanced by the cotreatment of quercetin and cisplatin in oral squamous cell lines were caspase-dependent. (A)** Tca-8113 cells were pretreated with quercetin (25, 50, 100 μM) for 2 h, followed by the addition of cisplatin (5 μg/ml) for an additional 24 h. Cell lysates were collected and subjected to Western blot analysis for the detection of pro-proteins and their cleavages of PARP, caspase-3, caspase-8, and caspase-9, respectively. **(B)** SCC-15 cells were pretreated with quercetin (100 μM) for 2 h, followed by the addition of cisplatin (5 μg/ml) for another 6, 12, 24 h. Cell lysates were collected and subjected to Western blot analysis for the detection of pro-proteins and their cleavages of PARP, caspase-3, caspase-8, and caspase-9. **(C)** Tca-8113 and SCC-15 cells were pretreated with pan-caspase inhibitor, Z-VAD-fmk (10 μM), for 30 min, before cells were incubated with the combination of cisplatin (5 μg/ml) and quercetin (100 μM), as mentioned above. The photos were taken under normal and inverted fluorescence microscope. The result was presented as the percentage of cells with evident nuclear condensation in 200 randomly selected cells. Western blot analysis was applied for the detection of cleavages of PARP, caspase-3, caspase-8 and caspase-9 (D for SCC-15 cells and A for Tca-8113 cells). The content of tubulin or GAPDH was used as a loading control. *p<0.05, in comparison to the untreated control; #p <0.05, in comparison to the combination of quercetin and cisplatin.

Caspase activation has an important function in both intrinsic and extrinsic apoptotic pathways. In this study, the expression of executor caspase protein caspase-3, initiator caspases upstream of caspase-3, caspase-8 in the death receptor pathway and caspase-9 in the mitochondrial pathway were all measured after treating Tca-8113 and SCC-15 cells with different drug doses and at different time points (**Figure 2A** and **2B**). The involvement of the caspase cascade in cell apoptosis induced by the co-treatment of quercetin and cisplatin was further examined by applying a pan-caspase inhibitor, Z-VAD-fmk. Consistently, pretreatment of Z-VAD-fmk efficiently inhibited PARP, caspase 3, 8 and 9 activation (**Figure 2A** and **2D**), and reversed the cell death (**Figure 2C**). Taken together, our results demonstrated that the combination of quercetin and cisplatin promote human oral squamous cells apoptosis, and that caspase was involved in this process.

### 3.3 NF-κB activation is suppressed by the combination of quercetin and cisplatin

**Figure 3:**
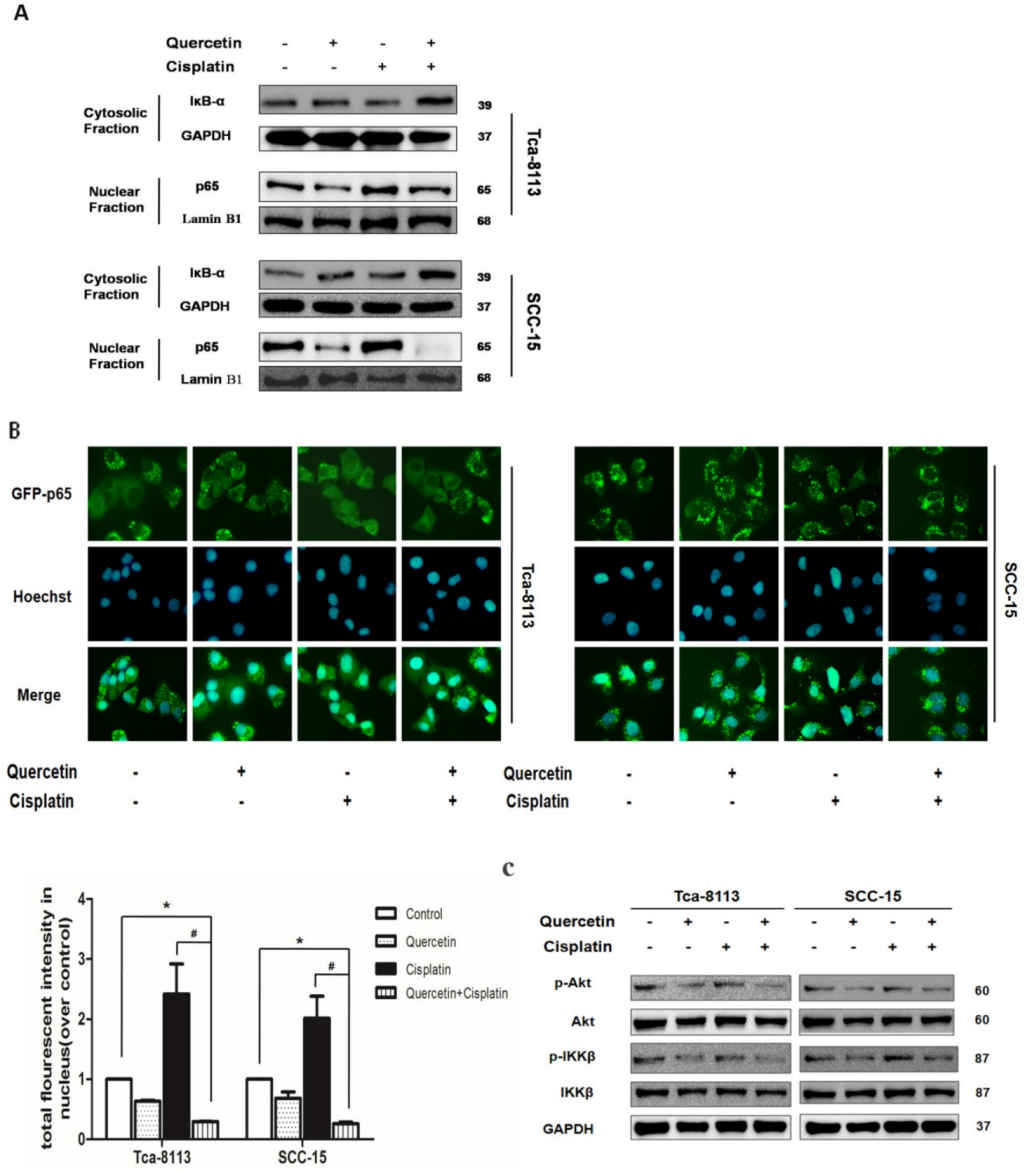
** Cotreatment of cells with quercetin and cisplatin suppressed NF**κ**B transcription activity. (A)** The effect of cotreatment on IκBa degradation and p65 nuclear translocation. Tca-8113 and SCC-15 cells were treated with or without quercetin (100 μM 2 h), followed with cisplatin (5 μg/ml) for 30 min. After treatment, cells were collected and nuclear and cytosolic extracts were prepared as previously described (*see the Materials and Methods section*). The levels of IκBa in cytosol and p65 in nuclear fraction were detected using Western blot. Tubulin and Lamin B1 served as the loading control for cytosolic and nuclear fractions respectively. **(B)** The translocation of p65 to nuclei was detected by immunofluorescence. Tca-8113 and SCC-15 cells were treated as mentioned above. Images of nuclear localization of p65 were photographed and the intensity of p65 fluorescence in nuclei was evaluated by Cellomics Toxinsight Reader. Hoechst 33342 staining was used to visualize the cell nuclei. Data obtained from three independent experiments are presented as means ± SD. *p < 0.05 vs untreated control; ^#^p< 0.05 vs combination of quercetin and cisplatin (one-way ANOVA with LSD'S test). (C) The effects of cotreatment on the activation of Akt and IKKβ. Tca-8113 and SCC-15 cells were treated with or without quercetin (100 μM 2 h) and then followed by the incubation in cisplatin (5 μg/ml) for 5 min. Western blot analysis was used to detect the total and phosphorylation level of Akt and IKKβ. GAPDH served as the loading control.

NF-κB transcription factors are homo- and heterodimeric complexes associated with tumorigenesis, including promoting cancer-cell proliferation and preventing apoptosis. The activity of NF-κB contributes to cisplantin resistance in OSCC 3. In canonical NF-κB pathway, the IKKβ subunit of the IκB kinase (IKK) complex is phosphorylated after stimulation, to promote ubiquitinates and the degradation of IκBα; then, NF-κB is activated by p65 nuclear translocation to initiate genes transcription 3. In order to understand the underlying mechanism responsible for the combinative effect of quercetin and cisplatin on apoptosis, we systematically examined their effects on NF-κB signaling pathways. As shown in **Figure 3A**, cisplatin treatment for 30 min promoted an increased translocation of p65 into nuclei in Tca-8113 and SCC-15 cells; while the cotreatment with quercetin significantly inhibited both IκBa degradation and subsequent p65 nuclear translocation. The suppression of p65 nuclear translocation was consistent with the results of IF. In addition, statistical differences in densitometric analyses of fluorescence in nuclei were observed between cisplatin alone and the combination group (**Figure 3B**). The upstream of NF-κB, IKKβ and Akt phosphorylation were also detected. As shown in **Figure 3C**, decreased expression of IKKβ and Akt were found.

### 3.4 Effects of quercetin and cisplatin on apoptotic protein xIAP regulated by NF-κB

**Figure 4:**
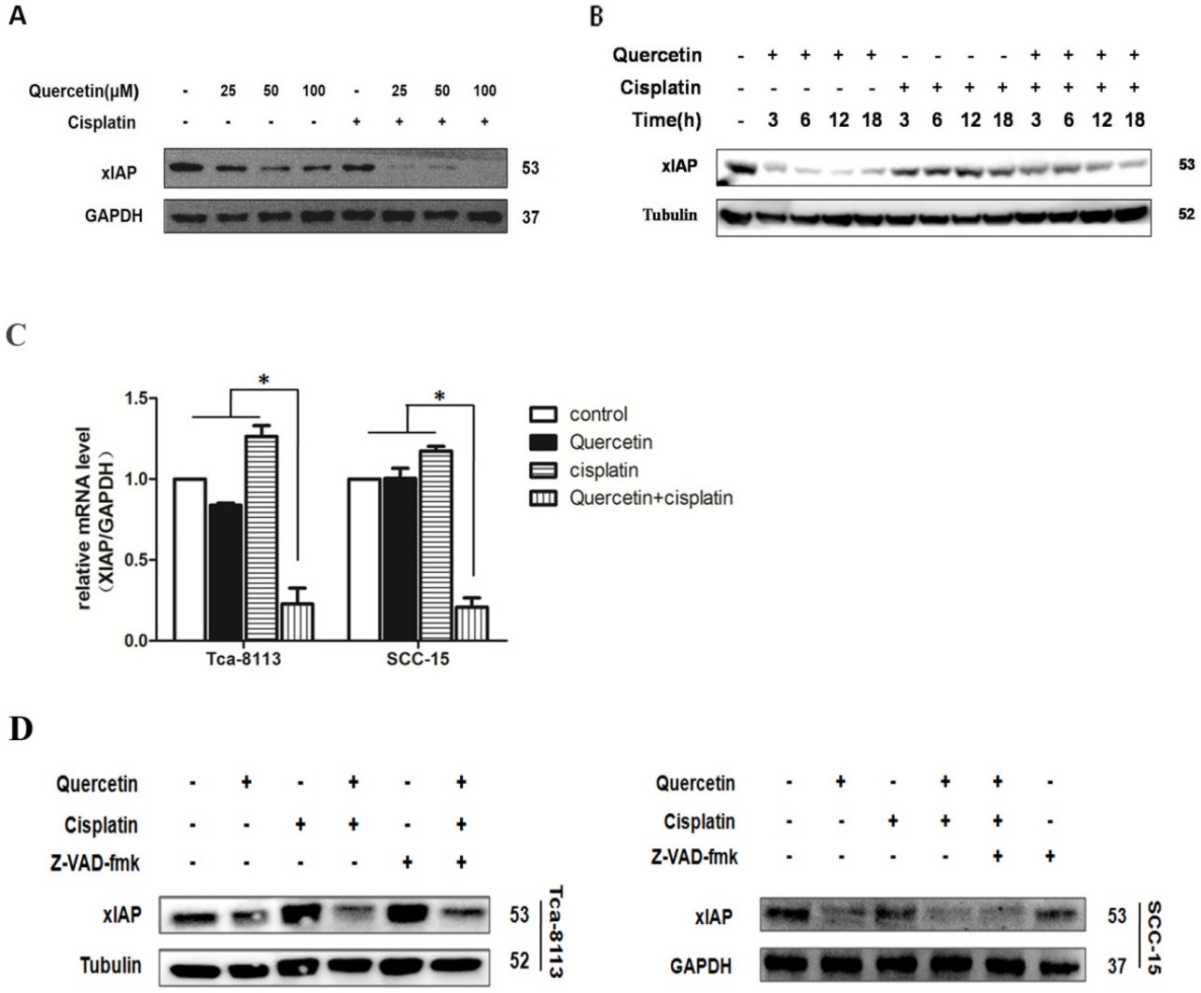
** Cotreatment of quercetin and cisplatin downregulated the xIAP expression. (A)** Quercetin downregulated xIAP in dose-dependent manner. SCC-15 cells were pretreated with quercetin (25, 50, 100 μM) for 2 h, followed by the addition of cisplatin (5 μg/ml) for another 24 h. Cell lysates were collected and subjected to Western blot analysis for the detection of xIAP. **(B)** Quercetin downregulated xIAP in time-dependent manner. Tca-8113 cells were pretreated with quercetin (100 μM) for 2 h, followed by the addition of cisplatin (5 μg/ml) for another 6, 12, 18 h. Cell lysates were collected and subjected to Western blot analysis for the detection of xIAP protein. **(C)** Tca- 8113 and SCC-15 cells were pretreated with pan-caspase inhibitor, Z-VAD-fmk (10 μM), for 30 min, before cells were incubated with the combination of quercetin (100 μM) and cisplatin (5 μg/ml) for 12h. Total RNA was extracted for RT-PCR to detect the expression of xIAP at mRNA level. GAPDH gene served as an internal control. *p < 0.05 vs control, quercetin or ciaplatin alone (one-way ANOVA with LSD'S test). **(D)** Tca-8113 and SCC-15 cells were pretreated with pan-caspase inhibitor, Z-VAD-fmk (10 μM), for 30 min, before cells were incubated with quercetin (100 μM) and cisplatin (5 μg/ml) for 24h. Cell lysates were collected and subjected to Western blot to analyze xIAP. The content of tubulin or GAPDH was used as a loading control.

Different anti-apoptotic genes, including inhibitors of apoptosis (IAPs), caspase-8/FADD (FAS-associated death domain)-like IL-1beta-converting enzyme (FLICE) inhibitory protein (c-FLIP) and Bcl-2 family regulate the anti-apoptotic function of NF-κB. Here, we further examined whether the co-treatment with quercetin and cisplatin affected the expression level of these genes. We found that the xIAP was significantly downregulated at both, protein level (**Figure 4A** and **4B**) and mRNA level (**Figure 4C**). As shown in **Figure 2A** and **2B**, cleavages implied the activation of caspase cascade induced by quercetin pretreatment, strongly suggesting that the upstream events above the activation of caspase-8 and caspase-9 were involved in this sensitization process. xIAP was the only member of IAPs able to directly inhibit both the initiation and execution phase of the caspase cascade; while, the inhibitors of pancaspase, caspase-8 and caspase-9 were not able to reverse the downregulation of xIAP caused by quercetin and cisplatin (**Figure 4D**), which further demonstrated that xIAP is the upstream apoptosis event.

### 3.5 The overexpression of xIAP protects the cells from apoptosis induced by quercetin and cisplatin

**Figure 5:**
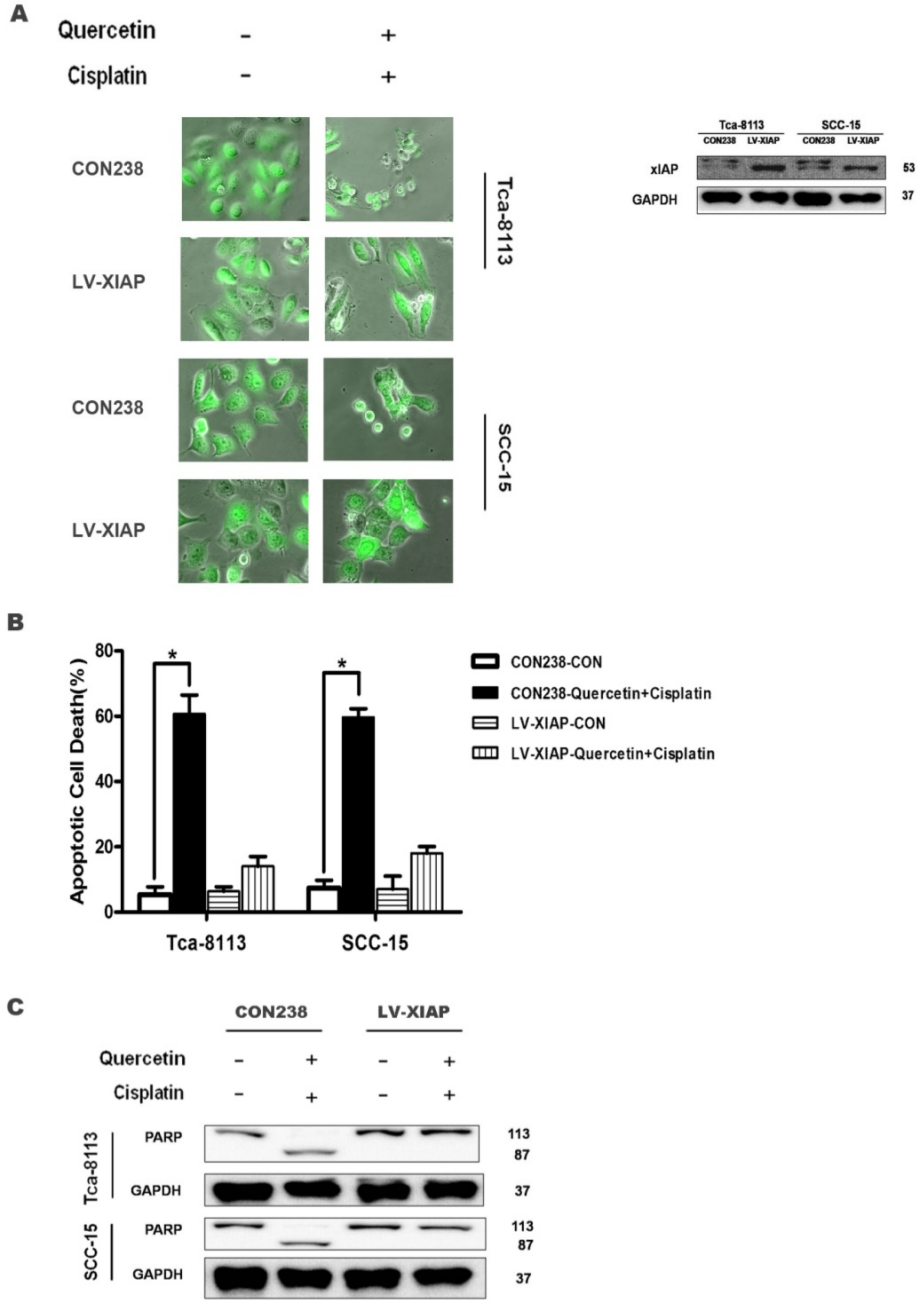
** Ectopic expression of xIAP could prevent apoptosis induced by the combination of quercetin and cisplatin. (A)** The Tca-8113 and SCC-15 cells were stably transfected with LV-XIAP or CON238 with EGFP; next, cell lysates were collected for Western blot analysis for the detection of xIAP transfection. After verification, cells were treated with quercetin (100 μM) and cisplatin (5 μg/ml) for 24h, and photos were taken under inverted fluorescence microscope. **(B)** Quantification of cell death by counting the percentage of dead cells among those transfected cells in total of randomly selected 200 transfected cells. Data are presented as means ± SD from three independent transfection experiments. *p < 0.05 vs untreated cells with control vector (CON238) (Student's t-test). **(C)** Cells were treated with the combination (pretreatment with quercetin 100 μM×2 h + 5 μg/ml cisplatin for another 24 h), and cell lysates were collected for Western blotting to detect PARP. GAPDH served as the loading control.

The above data collectively demonstrate that the reduced expression of anti-apoptotic gene xIAP may contribute to the combinative effect of quercetin and cisplatin on apoptosis. Here, we used a genetic approach to further establish the causative link between these events. The Tca-8113 and SCC-15 cells were stably transfected with LV-XIAP or CON238 with EGFP as a transfection marker. As shown in **Figure 5A**, strong green fluorescence was detected in the successfully transfected cells; in addition, the overexpression of antiapoptotic xIAP was found in those cells. As expected, following the co-treatments with quercetin and cisplatin, a majority of the cells transfected with CON238 died, while over-expression of xIAP offered protection against apoptotic cell death (**Figure 5A** and **5B**) and reversed changes of apoptotic hallmark proteins induced by quercetin and cisplatin (**Figure 5C**). This confirmed our earlier findings that such apoptosis was partially mediated by xIAP. These results further confirmed the role of xIAP in the combinative effect of quercetin and cisplatin.

### 3.7 xIAP have an important role in the anticancer effect induced by quercetin and cisplatin *in vivo*

**Figure 7:**
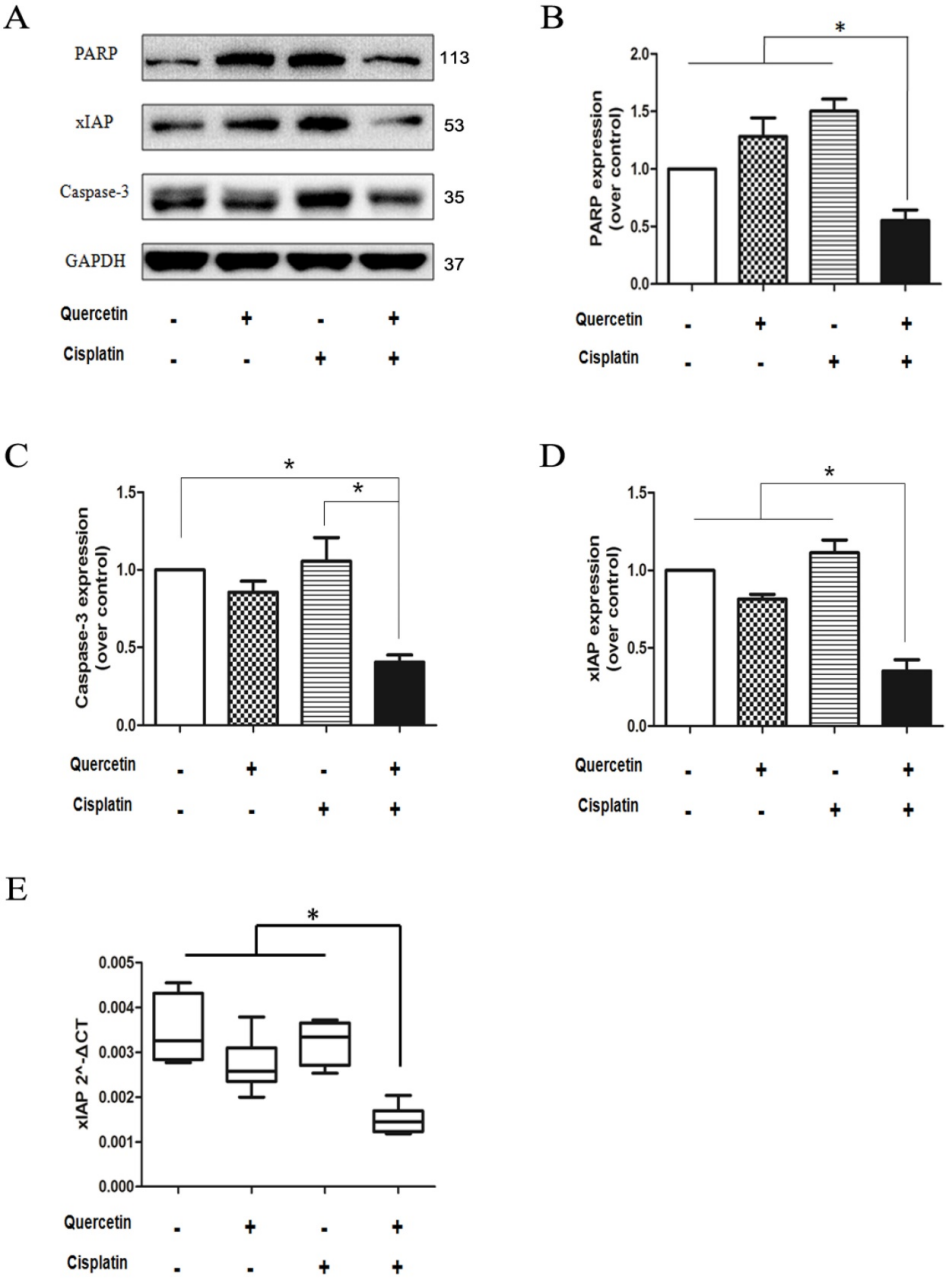
** The combination of quercetin and cisplatin downregulated xIAP expression *in vivo*. (A)** Cell lysates extracted from the excised tumor tissue were subjected to Western blot for the detection of caspase-3, PARP, and xIAP expression after the treatment mentioned above in Fig.6. GAPDH was used as a loading control. **(B-D)** The expression levels of PARP, caspase-3 and xIAP proteins of vehicle control, quercetin alone, cisplatin alone and the combinative group were quantified by densitometry and normalized to the internal control tubulin. **(E)** Total RNA was extracted from the excised tumor tissue for RT-PCR to detect the expression of xIAP at mRNA level. GAPDH was used as an internal control. The values were represented as means ± SD. *p < 0.05 vs vehicle control, quercetin or cisplatin alone (one-way ANOVA with LSD'S test).

Analysis of PARP and caspase-3 proteins (**Figure 7A**) from isolated tumor tissues revealed the consistent *in vitro* results, which implied that combination of quercetin and cisplatin could further enhance apoptosis in human OSCC cells. The density was quantified by densitometry. To explore the underlying mechanisms, the xIAP protein and mRNA were determined in tumor tissues using Western blot and RT-PCR. As shown in **Figure 7A** and **7E**, low level of xIAP was observed in the combination group, compared to the untreated control, quercetin or cisplatin alone, respectively. Such findings were consistent with *in vitro* cell culture experiments (**Figure 5**) and supported the notion that quercetin enhanced the therapeutic activity of cisplatin via down-regulation of xIAP.

## 4. Discussion

Currently, cisplatin-based chemotherapeutic regimens are used as adjuvant treatments against OSCC, especially for cancers with aggressive features. Nevertheless, resistance to cisplatin is a major limitation for its clinical application. Therefore, combined treatment with other sensitizing agents is an effective strategy to overcome cisplatin resistance 10. Herein, we provided experimental evidence that combination of quercetin and cisplatin may enhance the therapeutic potential for human OSCC. In addition, we found that the anticancer effect of quercetin and cisplatin is regulated through the inhibition of NF-κB activation, which leads to reduced expression of anti-apoptotic gene, xIAP, targeted by NF-κB. Our data revealed a novel function of quercetin and enhanced the value of quercetin as anti-cancer assistant agent.

It has been reported that quercetin induces selective activity of sensitizing cancer cells to radiotherapy and chemotherapy, but spares normal cells 11. Quercetin is described as a multi-target inhibitor with pleiotropic and synergistic effects in tumor cells, and has been reported to interfere with different targets 8. For example, quercetin alone or in combination with temozolomide or irradiation could induce apoptosis by suppressing the MAPK/ERK and PI3K/AKT signaling pathways in glioblastoma cells 11, 12. In addition, quercetin and its derivates have shown an anti-proliferative potential in bladder cancer by activating AMPK signaling pathway 13. Quercetin may also reduce ZD55-TRAIL mediated NF-κB activation and down-regulate its downstream targets thus sensitizing human hepatocellular carcinoma cells to apoptosis 14. Since it has long been a part of the human diet, quercetin is an ideal candidate to be combined with anticancer chemicals to improve their effectiveness.

In our study, quercetin or cisplatin alone have shown limited cytotoxicity in the OSCC cells. Yet, the combination of quercetin and cisplatin induced a significant cell death compared to untreated control, cisplatin alone or quercetin alone (Fig. 1A and 1B). Furthermore, our data revealed that the quercetin and cisplatin together promoted caspase-dependent extrinsic and intrinsic apoptosis of OSCC cell lines (Tca-8113 and SCC-15) *in vitro* (Fig. 1C, Fig. 2A and 2B) and *in vivo* (Fig. 7A-C).

NF-κB is an important signaling pathway involved in the pathogenesis and treatment of cancers 15. Compared to cisplatin alone, combined treatment with both quercetin and cisplatin could significantly increase the IκBα protein (IκBα inhibits the NF-κB transcription) in OSCC cell cytoplasm, and obviously reduce the p65 in the cell nucleus (Fig. 3A and 3B), which further suggests that NF-κB activation is suppressed by the combination of quercetin and cisplatin.

NF-κB, often driven by the IKK complex that consists of IKKα, β, and γ subunits, can phosphorylate IκBα and lead to its ubiquitination and proteasomal degradation. Upon its activation, Akt acts as an upstream regulatory factor of IKK and utilizes IKK to activate NF-κB transactivation potential and to induce RelA/p65 phosphorylation 16-20. Several studies have shown that Akt directly phosphorylates IKKα. Nonetheless, some studies have also indicated that IKKβ is a substrate of Akt in epidermal cells 21, HTLV-1-transformed cells 22, breast cells 23, and lymphoma cells 24. It has been reported that quercetin treatment could inhibit Akt (S473) phosphorylation in many cancer cells 11, 12, 25. Our data also confirmed that phosphorylated Akt and IKKβ significantly decreased when the cells were treated with quercetin alone or with combined quercetin and cisplatin treatment (Fig. 3C). This data suggested that the inhibition of Akt and IKKβ phosphorylation was an essential process for the suppression of NF-κB through prevention of IκBα degradation, which further led to NF-κB suppression.

The expression of NF-κB-dependent genes such as FLIP, xIAP, Bcl-2, Bcl-xL and COX-2 is known to be associated with chemoresistance. X-linked inhibitor of apoptosis (xIAP) is a member of the inhibitors of apoptosis protein family (IAP) and the only member of this family that can directly inhibit both the initiation and execution phase of the caspase cascade in order to mediate the controlled demise of malignancy. Elevated expression of xIAP protein has been reported in almost all of 60 human cancer cell lines 16, 26, 27 and high expression levels of xIAP have shown to be correlated with chemoresistance and poor prognosis in several types of cancer 28, 29. Consequently, xIAP has become a promising target for research into novel antitumor drugs. According to the structural characterization of xIAP, various inhibitors have been explored so far. In this study, compared with cisplatin alone, the effect of combined treatment of quercetin and cisplatin on xIAP protein has been associated with time- and dose-dependent decrease *in vitro* (Fig. 4A and 4B) and *in vivo* (Fig. 7A and 7D). Down-regulation of xIAP protein is upstream of apoptosis, because this effect on xIAP can be reversed by Z-VAD-fmk (Fig. 4D). The down-regulation of xIAP at mRNA level has suggested that the usage of quercetin and cisplatin together could influence xIAP through transcription (Fig. 4C and 7E). Exogenous expression of xIAP can prevent apoptosis induced by the combination of quercetin and cisplatin (Fig. 5). All these data confirmed that quercetin synergized with cisplatin to induce the apoptosis in OSCC cells by downregulating xIAP through NF-κB signaling pathway.

In summary, our study verified that quercetin is an efficient synergic anticancer agent in OSCC *in vitro* and *in vivo*. By suppressing NF-κB, quercetin downregulates xIAP, and sensitizes the cisplatin-induced apoptosis in OSCC. The current study provides a base for the usage of quercetin as an assistant agent for chemotherapy in OSCC, while the mechanism involving Akt-IKKβ-NF-κB-xIAP axis pathway may be translated as a future clinical strategy for OSCC treatment.

## Figures and Tables

**Figure 6:**
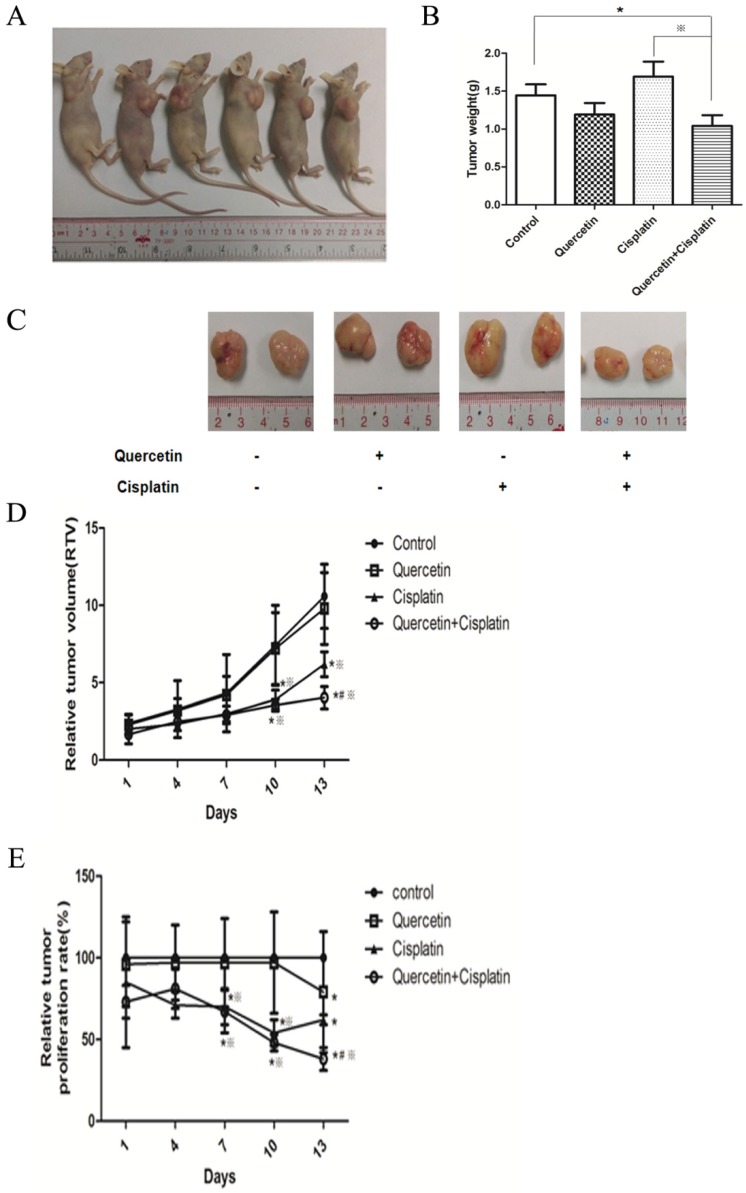
** Anticancer effect of quercetin and cisplatin in a tumor xenograft mouse model. (A)** BALB/c nude mice were inoculated with Tca-8113 cells as described in the Materials and Methods section. After the implanted tumor cells formed palpable nodules, mice were pretreated with quercetin (50 mg/kg body weight, ip everyday) for one week, followed by cisplatin treatment (2 mg/kg body weight, ip three times a week) for another two weeks. The experimental groups included quercetin group (50 mg/kg body weight, ip everyday), cisplatin group (2 mg/kg body weight, ip three times a week), combination group and vehicle control group (n=6). **(B)** After treatment, Tca-8113-xenografted tumors were collected and analyzed in *ex vivo*; tumor weights were recorded and the representative photos of excised tumor tissue were taken (×200). **(C)** During the procedure, tumor volumes were measured at each time point (every three days). Then the relative tumor volume (RTV) **(D)** and the relative tumor proliferation rate **(E)** were calculated. The error bars represent the standard error of a group. *p < 0.05 vs vehicle control group; ^#^p <0.05 vs cisplatin group;^※^p <0.05 vs quercetin group.
